# The Effects of External Jugular Compression Applied during Head Impact Exposure on Longitudinal Changes in Brain Neuroanatomical and Neurophysiological Biomarkers: A Preliminary Investigation

**DOI:** 10.3389/fneur.2016.00074

**Published:** 2016-06-06

**Authors:** Gregory D. Myer, Weihong Yuan, Kim D. Barber Foss, David Smith, Mekibib Altaye, Amit Reches, James Leach, Adam W. Kiefer, Jane C. Khoury, Michal Weiss, Staci Thomas, Chris Dicesare, Janet Adams, Paul J. Gubanich, Amir Geva, Joseph F. Clark, William P. Meehan, Jason P. Mihalik, Darcy Krueger

**Affiliations:** ^1^Division of Sports Medicine, Cincinnati Children’s Hospital Medical Center, Cincinnati, OH, USA; ^2^The Human Performance Laboratory, Division of Sports Medicine, Cincinnati Children’s Hospital Medical Center, Cincinnati, OH, USA; ^3^Department of Pediatrics, College of Medicine, University of Cincinnati, Cincinnati, OH, USA; ^4^Department of Orthopaedics, University of Pennsylvania, Philadelphia, PA, USA; ^5^The Micheli Center for Sports Injury Prevention, Waltham, MA, USA; ^6^Department of Orthopaedic Surgery, University of Cincinnati, Cincinnati, OH, USA; ^7^Pediatric Neuroimaging Research Consortium, Cincinnati Children’s Hospital Medical Center, Cincinnati, OH, USA; ^8^Department of Athletic Training, Division of Health Sciences, Mount St. Joseph University, Cincinnati, OH, USA; ^9^Department of Neurosurgery, NorthShore University Health Systems, Evanston, IL, USA; ^10^Division of Biostatistics and Epidemiology, Cincinnati Children’s Hospital Medical Center, Cincinnati, OH, USA; ^11^ElMindA, Ltd., Herzliya, Israel; ^12^Division of Radiology, Cincinnati Children’s Hospital Medical Center, Cincinnati, OH, USA; ^13^Department of Psychology, Center for Cognition, Action and Perception, University of Cincinnati, Cincinnati, OH, USA; ^14^Department of Electrical and Computer Engineering, Ben Gurion University of the Negev, Beer Sheva, Israel; ^15^Department of Neurology, College of Medicine, University of Cincinnati, Cincinnati, OH, USA; ^16^Division of Sports Medicine, Boston Children’s Hospital, Boston, MA, USA; ^17^Department of Pediatrics and Orthopedics, Harvard Medical School, Boston, MA, USA; ^18^Department of Exercise and Sport Science, Matthew Gfeller Sport-Related Traumatic Brain Injury Research Center, University of North Carolina, Chapel Hill, NC, USA; ^19^Division of Neurology, Cincinnati Children’s Hospital Medical Center, Cincinnati, OH, USA

**Keywords:** head injury, pediatric traumatic brain injury, traumatic brain injury, EEG

## Abstract

**Objectives:**

Utilize a prospective *in vivo* clinical trial to evaluate the potential for mild neck compression applied during head impact exposure to reduce anatomical and physiological biomarkers of brain injury.

**Methods:**

This project utilized a prospective randomized controlled trial to evaluate effects of mild jugular vein (neck) compression (collar) relative to controls (no collar) during a competitive hockey season (males; 16.3 ± 1.2 years). The collar was designed to mildly compress the jugular vein bilaterally with the goal to increase intracranial blood volume to reduce risk of brain slosh injury during head impact exposure. Helmet sensors were used to collect daily impact data in excess of 20 g (games and practices) and the primary outcome measures, which included changes in white matter (WM) microstructure, were assessed by diffusion tensor imaging (DTI). Specifically, four DTI measures: fractional anisotropy, mean diffusivity (MD), axial diffusivity, and radial diffusivity (RD) were used in the study. These metrics were analyzed using the tract-based Spatial Statistics (TBSS) approach – a voxel-based analysis. In addition, electroencephalography-derived event-related potentials were used to assess changes in brain network activation (BNA) between study groups.

**Results:**

For athletes not wearing the collar, DTI measures corresponding to a disruption of WM microstructure, including MD and RD, increased significantly from pre-season to mid-season (*p* < 0.05). Athletes wearing the collar did not show a significant change in either MD or RD despite similar accumulated linear accelerations from head impacts (*p* > 0.05). In addition to these anatomical findings, electrophysiological network analysis of the degree of congruence in the network electrophysiological activation pattern demonstrated concomitant changes in brain network dynamics in the non-collar group only (*p* < 0.05). Similar to the DTI findings, the increased change in BNA score in the non-collar relative to the collar group was statistically significant (*p* < 0.01). Changes in DTI outcomes were also directly correlated with altered brain network dynamics (*r* = 0.76; *p* < 0.05) as measured by BNA.

**Conclusion:**

Group differences in the longitudinal changes in both neuroanatomical and electrophysiological measures, as well as the correlation between the measures, provide initial evidence indicating that mild jugular vein compression may have reduced alterations in the WM response to head impacts during a competitive hockey season. The data indicate sport-related alterations in WM microstructure were ameliorated by application of jugular compression during head impact exposure. These results may lead to a novel line of research inquiry to evaluate the effects of protecting the brain from sports-related head impacts via optimized intracranial fluid dynamics.

## Introduction

The World Health Organization projected that traumatic brain injury (TBI) will rank as the third leading cause of global disease and injury by 2020 (1). The annual cost is over $60 billion in the United States alone (2) for the diagnosis, treatment, and management of TBI. These monetary costs, combined with the poor long-term prognosis and heightened awareness of the potential long-term sequelae of multiple concussions and subconcussive blows, indicate that innovative prevention strategies are needed to reduce the significant morbidity associated with concussion and mild TBI that result from sports-related head impacts (3).

The most common historical solutions to this growing epidemic have been in the innovation and improvement of helmet design, rule changes, and restricted participation in high-risk sports (4, 5). These advancements, however, have not reduced concussion incidence or reported symptoms (4–6). Moreover, this focus on helmets does little to address the concussion epidemic in non-helmeted sports (2). In fact, helmets, and other touted protective gear (5, 7), do not address the underlying mechanism of concussion injury – the dynamic forces that cause movement of the brain inside the cranium, commonly referred to as *slosh* (8, 9). Thus, neither rule changes or invoking fear of TBI to reduce participation in certain sports has diminished the incidence of concussions and neither is an acceptable long-term solution (10).

The biomechanical forces imparted to the brain during sports-related collisions can result in a wide range of trauma, from asymptomatic impacts and cerebral concussion to diffuse axonal injuries. In hockey and other collision sports, the majority of impacts fall between 20 and 25 g of linear acceleration, although hits from 50 to 75 g are common (11–14). When exposed to impact, the brain risks slosh-induced injury as tissues of differing density accelerate/decelerate at diverse rates, which induces shear forces. Neuropathological findings suggest that even subconcussive impacts to the head can lead to persistent cognitive impairments in various neuro-cognitive domains, including attention, memory, and executive function (15).

**Figure 1:**
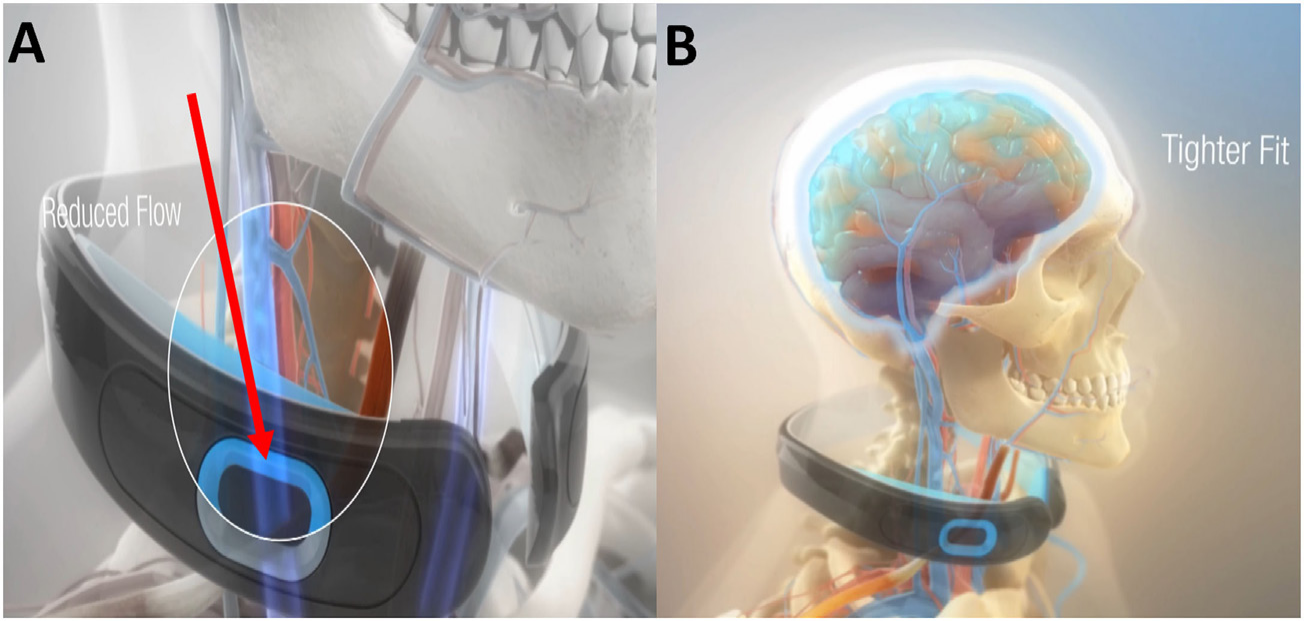
**The Q-Collar designed to facilitate the actions of the compressive effect of the omohyoids to reduce blood outflow of the brain (A) and produce a tighter fit of the brain within the cranium (B)**.

Given the common range of impact levels in humans and their associated sequela (16), it was contemplated how a ram can survive a 500 g collision directly to the head (17), and similarly how woodpeckers’ brains tolerate repeated 1200 g impacts? (18) Observations suggest that both may have evolved the ability to modulate their intracranial pressure/volume via jugular vein impedance (19). Researchers have suggested that a similar protective mechanism to reduce brain slosh may exist in humans (9, 19, 20). Contraction of the omohyoid neck muscles can gently indent and constrain the jugular veins slowing the outflow of blood from the brain (21). Compression of the jugular vein in humans can also result in increased volume of the venous capacitance vessels of the cranium (22). Based on these physiological models, filling the compensatory reserve volume (23) within the cranium is hypothesized to create a cradling effect that increases brain stiffness (24), reduces slosh of the brain inside the skull, and reduces risk of brain injury. In preclinical models for TBI, we have demonstrated significant reductions in axonal amyloid precursor proteins, degenerative neurons, reactive astrocytes, and microglial activation in collared vs. no-collar rodents (8, 19). Drawing from these predicate studies, a collar has been developed for humans to gently facilitate the actions of the omohyoid muscular complex in modulating intracranial blood volume to produce a tighter fit of the brain within the cranium (Figures 1A,B).

The current study presents a novel, *in vivo* clinical trial evaluating the Q-Collar during sport (i.e., hockey) to test its effect in ameliorating both neuroanatomical and neurophysiological biomarkers. Specifically, anatomical white matter (WM) integrity was evaluated using diffusion tensor imaging (DTI), and physiological changes in functional connectivity reflecting the spatiotemporal dynamics of brain network responses were assessed using electroencephalography (EEG) event-related potentials (ERPs). We hypothesized that the collar device would significantly reduce the change in WM integrity and brain network neurophysiology relative to non-collar controls.

## Materials and Methods

### Study Participants

The Cincinnati Children’s Hospital Medical Center Institutional Review Board approved the data collection procedures and consent forms. IRB approval number is IRB # 2014-5009 (Clinical Trials.gov #: NCT02271451). Nineteen healthy male varsity level high school hockey players were recruited from Southwest Ohio, 17 of them were enrolled. Parents, guardians and athletes provided informed consent and assent prior to participation in the study. Normal, healthy volunteers who were able to provide written consent and were on the varsity hockey team roster were included in the study. Exclusion criteria for study participation included history of neurological deficits, previous cerebral infarction, severe head trauma, known increased intracerebral pressure, metabolic acidosis or alkalosis, glaucoma (narrow angle or normal tension), hydrocephalus, recent (within 6 months) penetrating brain trauma, known carotid hypersensitivity, central vein thrombosis, known airway obstruction, or seizure disorder. Of the 17 subjects enrolled, two participants had contraindications for MRI imaging, one with upper and lower dental braces and one with an existing metal implant from a prior surgery. In addition, evaluation of the athlete with the indwelling metal plate did not provide usable EEG data and, thus, was excluded from the study. One athlete chose to withdraw due to irritation upon wearing the collar at the first practice. This left 15 subjects for final analysis (aged 16.3 ± 1.2 years): 15 with EEG outcomes and 14 DTI. The 15 athletes were randomly assigned to one of two groups: (1) device wearing during the first half of the season or (2) non-device wearing during the first half of the season.

### Study Design

**Figure 2:**
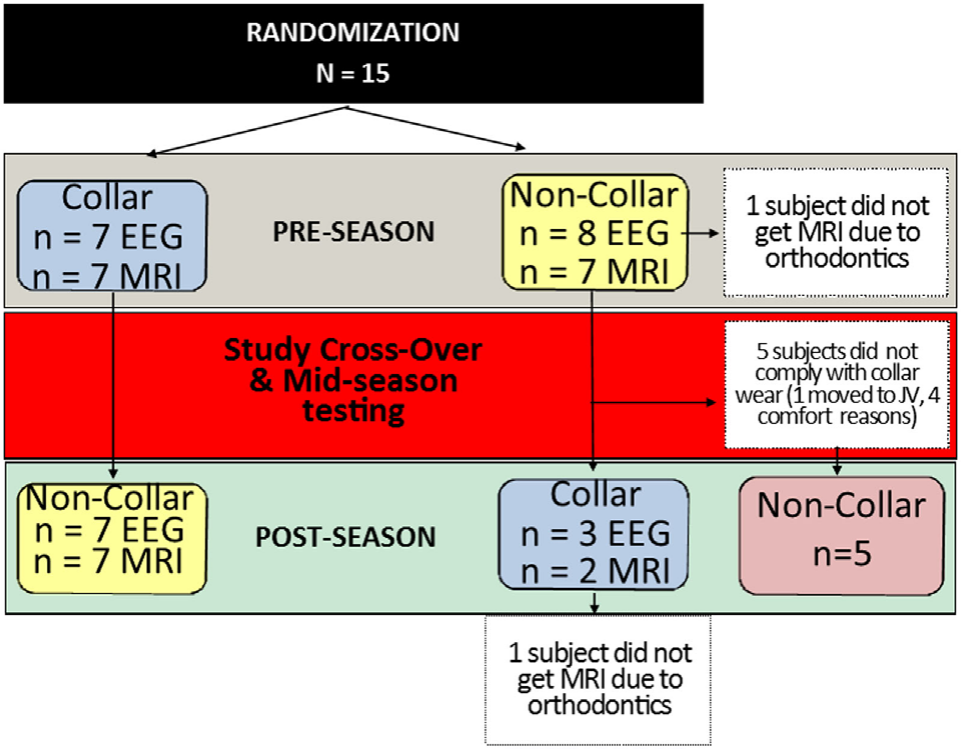
**Study subject allocation following randomization**. Subjects randomly assigned to the collar (*n* = 7) or no-collar group (*n* = 8) for the first half of the season were crossed-over at the mid-season time point. The number of subjects who completed each test (EEG and MRI) at each time point is indicated in the blue and yellow boxes. In the second half of the season, five subjects did not comply with wearing the collar, which created a second “No-Collar” group in the latter part of the season, as indicated in the red box.

This investigation was planned using a 2 × 2 cross-over study design; however, after the first phase of the study, more than half of the subjects who were scheduled to switch from the no collar to collar condition were not compliant, leaving only three subjects to comply with the collar use in the second half of the season (Figure 2). Given the non-compliance, the small number of subjects who complied with the protocol following cross-over, and the limited wash-out period, an intention to treat (ITT) analysis that included data from the mid- to post-season period was deemed not to be valid. Thus, we conducted analyses that were determined the most appropriate for this preliminary project, and only compared and presented the results of longitudinal assessments of data collected at baseline and mid-season (first period of hockey season, approximately 2 months) between the collar and no-collar groups in the current manuscript.

### Incidental Findings

On pre-season imaging, one athlete demonstrated an area of volume loss and encephalomalacia involving the left mesial temporal lobe and hippocampus with hemorrhagic staining; likely representing a remote insult. Clinical MRI was performed, including MR angiography that demonstrated no other baseline abnormality or definite etiology. The athlete was cleared by his neurologist for full participation in the study, and no changes were noted on subsequent mid-season imaging. One athlete demonstrated internal echoes within the left internal jugular vein (IJV) on pre-season sonography (without compressibility). Follow-up clinical CT and Doppler ultrasound were performed showing the IJVs to be normal without filling defects or thrombi. Subsequent Doppler ultrasound was performed and still showed increased internal echoes but with normal and complete compressibility. No further work-up was needed and the athlete was cleared for full participation in the study.

### Instrumentation and Procedures

Testing sessions were completed at three different time points: (1) pre-season (2) mid-season, and (3) post-season. Each testing session consisted of two visits: (1) laboratory testing with EEG and (2) MRI brain imaging that took place within ~1–2 days from the paired laboratory testing visit. The laboratory testing visit was completed at the Human Performance Laboratory in the Division of Sports Medicine at Cincinnati Children’s Hospital Medical Center (CCHMC). MRI testing was completed at the Imaging Research Center at CCHMC and consisted of a pre-imaging screening questionnaire to ensure each subject had no contraindications for MRI such as cochlear implants, implanted medical devices, etc. Imaging sequences included 3D T1 anatomic, DTI, and susceptibility weighted imaging (SWI), MR scans were acquired on a 3T Phillips scanner (Phillips Achieva, Phillips Medical Systems, Amsterdam, Netherlands) using a 32-channel head coil. In the present study, only DTI and SWI outcome measures were included in the report. Pre-season (baseline) testing took place prior to the start of competitive play. Mid-season testing was completed halfway through the season, while post-season testing took place after competitive play (including post-season play) was complete. The average time between testing was 63.8 ± 9.8 (range = 47–76) days for the pre- to mid-season phase and 77.2 ± 6.2 (range = 70–92) days for the mid- to post-season phase, which also included post-season tournament play. The average number of days between the last mid-season competition exposure and mid-season test sessions was 2.9 ± 1.8 (range = 1–6) days. Study visits were scheduled in coordination with subject, laboratory, and MR scanner availability. During the pre- to mid-season phase, there were 17 practices and 15 regular season games. During the second phase, including post-season tournaments, there were 26 practices and 21 regular or tournament games.

### MRI Data Acquisition

The DTI data were acquired with a spin echo planar sequence with the following specifications: TR/TE = 9000/83 ms; FOV = 256 mm × 256 mm, matrix = 128 × 128, in-plane resolution = 2 mm × 2 mm; slice thickness = 2 mm; 72 slices. Diffusion weighed images were acquired along 61 non-collinear directions with 7 no diffusion weighed images (b0 = 1000 s/mm^2^). All anatomical imaging (3D T1-weighted and SWI sequences) was evaluated by the same board certified neuroradiologist (JLL) blinded to treatment group assignment, at each imaging time point. Abnormal findings were reported to the study investigators, subject, and parents as per study protocol.

### MRI Data Processing and Analysis

Diffusion tensor imaging data were processed with the Functional MRI of the Brain (FMRIB) Software Library (FSL) software package (www.fmrib.ox.ac.uk/fsl). In FSL, skull stripping was performed using the brain extraction tool (BET) function. Eddy current and head motion artifact were corrected in FSL by aligning diffusion weighted images to the first b0 image with an affine transformation with 12 degrees of freedom. The following four commonly used DTI measures were calculated using standard methods: fractional anisotropy (FA), mean diffusivity (MD), axial diffusivity (AD), and radial diffusivity (RD) (25). The tract-based Spatial Statistics (TBSS) approach was used in the image analysis in the present study (26). This is a method developed to ameliorate the registration error at the boundary of narrow WM fiber bundles, a common source of error in voxel-based style analysis. Studies have shown that TBSS can effectively reduce the granularity and improve accuracy during the normalization. We followed standard TBSS analysis steps summarized briefly as follows: (1) after DTI scalar maps were generated, FA maps from all subjects were first aligned via a non-linear transformation to determine a target image that was closest to the mean of the FA maps in the study; (2) the target image was aligned to Montreal Neurological Institute (MNI) space using affine registration; (3) individual FA map was registered into the MNI space based on the combined transformation; (4) all the aligned FA maps were averaged to generate a mean FA and then thresholded at FA > 0.2 to create a mean FA skeleton, which represented the WM tracts most common to all the subjects; (5) FA maps from individual subjects were projected onto the skeleton with the FA values determined via a special algorithm for local maximum FA; and (6) the MD, AD, and RD maps were projected to the skeleton using the TBSS_non_FA function in FSL based on the same overall transformation as calculated in the processing of FA maps. The group statistical analysis was conducted only in the WM skeleton with the individual projected DTI maps as input and the skeleton as mask, thus restricting the analysis to determine the major WM tracts that are common to all subjects. In association with the current study, we explored the potential physiological effects of exercise and time on DTI measures in the absence of head impact exposure. To evaluate the stability of DTI measures, we captured a sub-sample of matched athletes from the same school competing in track and field. The track athletes were assessed using identical MR sequences to the current sample of hockey periods over a similar time period (data not shown). The results indicated no significant changes in DTI measures, for the specific DTI outcome measured, between baseline and follow-up.

### EEG Data Acquisition and Analysis

Each study subject performed an auditory oddball paradigm including a series of tones (*N* = 400) containing 80% standard tones (2000 Hz) and 10% target tones (1000 Hz). The remaining 10% of sounds were environmental sounds (i.e., a telephone ring, dog bark, etc.; the novel sound). During this task, sounds were presented at a fixed rate of 1 every 1.5 s. The subject’s task was to respond to the target sound by pressing a button. The Oddball task took approximately 12 min to complete. For all tasks, EEG channels were sampled at 250 Hz.

Post-processing of the EEG data consisted of a brain network activation (BNA) (27–31) analysis for the data measured via a standard high-density 64- or 128-electrode wet EEG cap fitted to the participant’s head. As part of the BNA preprocessing, all data were interpolated to a 64-electrode framework. For the Oddball task data, epoched segments (200 ms pre-stimulus to 1200 ms post-stimulus), using the 200 ms pre-stimulus interval, were averaged separately for the “frequent/standard,” “target,” and “novel” sounds. The EEG signals were recorded and cleaned by standard procedures, band pass filtered into overlapping physiological frequency bands [delta (0.5–4 Hz), theta (3–8 Hz), alpha (7–13 Hz), and beta (12–30 Hz)], cut into epochs demarking pre- and post-stimulus onset times, and averaged to align with ERPs. The data were reduced into a set of discrete points that denote local extrema for each band, and the resulting latencies and amplitudes were input into the BNA algorithm (28–30).

Detailed descriptions of the BNA analysis method are published elsewhere (27–29, 31). Briefly, the BNA analysis involves two independent processes: (1) a group-level pattern recognition process used to generate the characteristic group’s network and (2) a single-subject level similarity evaluation process in which a single subject is compared to the reference brain network model (RBNM). The degree of similarity with the RBNM is measured by the BNA score (27–29, 31). The coordinated activity in time, location, and frequency depicted in the RBNM implies that the spatiotemporal functional dependencies of regional neuronal activities in different frequency bands represent functional links common to and characteristic of a specific population (e.g., healthy controls, concussed patients). The RBNM serves as an integral part of the BNA algorithm, namely as a consistent electrophysiological template to assess the degree of congruence in terms of the network activation pattern between the single subject and the group’s pattern. The result is a BNA Score – a measure of similarity of Δt of a single subject compared to the Δt of all pairs of events in the RBNM, reflecting the time lagged dynamics of the co-occurrence between events. The BNA Score ranges between 0 (no similarity to the RBNM) and 100% (identical to RBNM). Thus, the higher the BNA Score the greater the similarity to the RBNM. Here, we report the absolute change in BNA Score (or BNA Change Score) between two visits (i.e., mid-season minus baseline).

### Neck Ultrasound Evaluation and Collar Fitting

**Figure 3:**
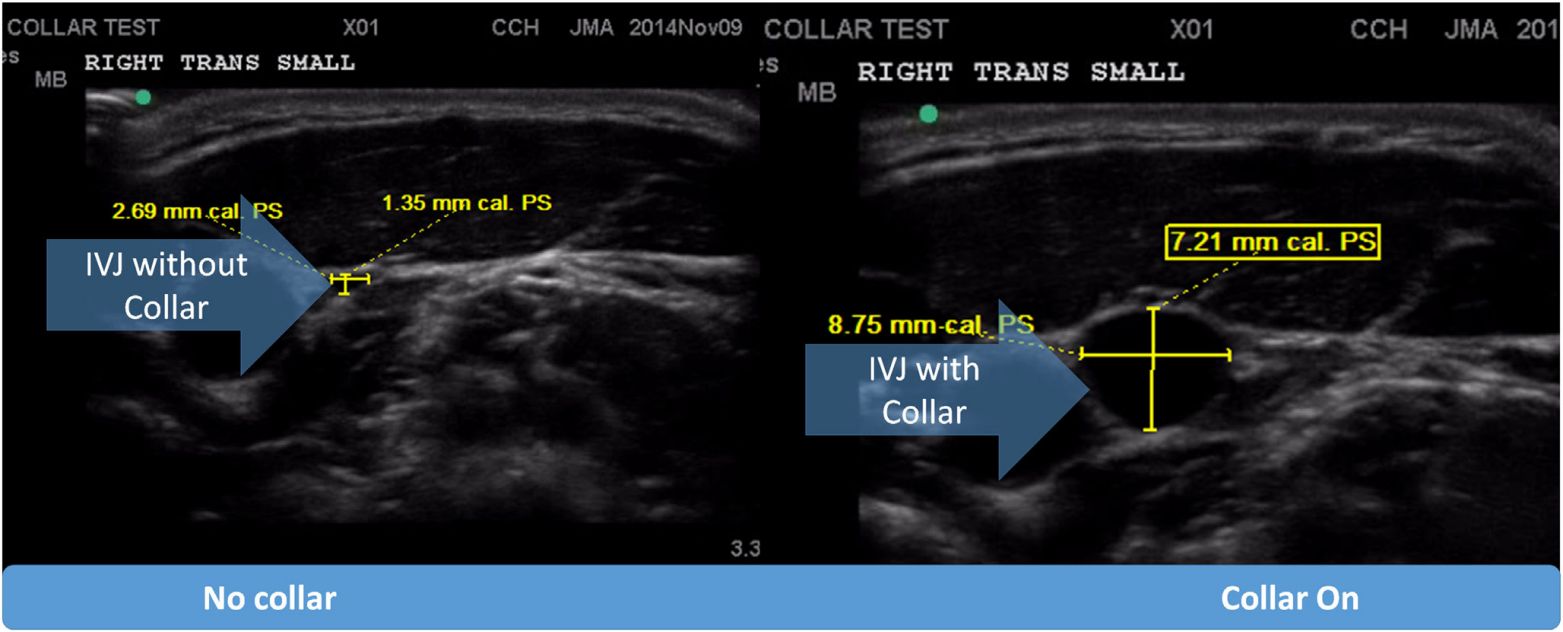
**Pictorial representation of internal jugular vein (IJV) response via dilation to the application of the collar device**.

The neck collar was designed to mildly compress the jugular vein bilaterally to increase intracranial blood volume in order to reduce risk of brain slosh injury during head impact exposure. The neck collar device is made of the following: outer collar – Urethane Thermoplastic elastomers (durometer 80 Shore A), an inner collar – Urethane Thermoplastic elastomers (durometer 50 Shore A), and an insert – Rigid Urethane, glass filled (Figures 1A,B). The individual collar size was determined from the measured user neck circumference, and validated in use by both measuring the spacing of the collar tips (1.25″–2.5″) and visual evidence of ultrasound IJV dilation. At the initial fitting of the collar, a registered vascular technologist (JA) utilized ultrasound to ensure that the proper collar and IJV responses (e.g., visual evidence of IJV dilation superior to collar) occurred as prescribed (Figure 3). All ultrasonography procedures and measurements were performed by a single sonographer and the images and video clips were acquired on a SonoSite M-MSK unit (SonoSite Inc-US, Bothell, WA, USA), using the HFL 50, 15–6 MHz linear transducer. During testing, the study participants sat upright, facing forward. Ultrasound coupling gel was applied to the lateral neck, and the right side common carotid artery and IJV were identified in the transverse plane, verifying normal anatomy. A collar was then placed in proper position around the subject’s neck. To further evaluate responsiveness to the collar, a 15 s video clip was obtained, recording the collar in proper position, the collar opened away from the neck, and the collar returned to its proper position. The IJV metrics were measured at their largest transverse dimensions upon initial placement of the collar, and then at the smallest transverse dimension when the collar was opened, and again after replacing the collar back on the neck. The collar imparts a gentle and continuous pressure to the area just above the omohyoid muscles. As there would be no way to know when an impact would occur, this continuous impedance serves to provide the proposed physiological response during the entire session (practice or game) in which the athlete may be exposed to head impacts (both intended and unintended). Ultrasound evaluations were taken to ensure that this rapid response to jugular compression occurred in the study participants. The Queckenstedt literature indicates that jugular compression fills the intracranial compensatory reserve in 0.5 s (20).

### Head Impact Surveillance

An athletic trainer affiliated with the high school attended every practice and game and provided head impact surveillance. Head accelerations were recorded using GForceTracker accelerometers (GForceTracker, Markham, ON, Canada) secured inside the back of each hockey helmet. With the exception of goalies (who wore specialized headgear), all subjects were provided with standardized headgear (Bauer RE-AKT, Exeter, NH, USA) and mouth guards (Nike, Beaverton, OR, USA). Accelerometers were placed in the same location and orientation inside each helmet. Goalies had accelerometers placed on an exterior portion of the helmet in the same location as teammates. During each practice and game, the athletic trainer activated the accelerometers and monitored the real-time transmission of impact data displayed on a laptop via telemetry. The start and end times of each exposure were recorded, and only accelerations recorded within that time window were aggregated from each participant’s accelerometer data. At the conclusion of each practice session or game, the accelerometers were turned off and all data were downloaded to the study database. The accelerometers were removed from the helmets and recharged twice weekly. The accelerometers were programed to record data only when experiencing accelerations above a 10 g threshold and acceleration data were collected at 3000 Hz. Prior to the initial exposure (i.e., first practice), each accelerometer was calibrated according to device specifications and relative to the eventual placement of the sensor in each helmet.

### Safety and Tolerance of Collar

Throughout the season, the school’s athletic trainer monitored the compliance of wearing the collar at each practice and game. For the pre- to mid-season period, there was a reported 98.3% compliance rate of collar use in the intervention group. After the cross-over from the mid-season, four of the seven players assigned to the collar condition in the second half of the season did not want to change equipment or reported enough irritation, e.g., perception of pressure on the neck, to cause them not to wear it for all exposures. One player reported feeling slightly nauseated after wearing the collar. Another player felt the collar made head turning difficult, yet he continued the season with the collar on. There was no evidence of acquired intracranial hemorrhage or gross brain injury on the mid-season and post-season imaging in any participant. No detrimental effect was found in collar wearing on EEG measures. At the end of the season, participants completed a questionnaire regarding the tolerance and acceptance of the collar while playing hockey and the responses were qualitatively assessed relative to acceptance and tolerance of collar wear.

### Statistical Analysis

The data indexing the number of total impacts was subjected to a Box–Cox natural log (Ln) transformation to alleviate extreme positive skewness observed in the data (32), while the total experienced *g*-forces were subjected to a simple Ln transformation due to moderate positive skewness. Separate independent *t*-tests were then conducted to compare the collar and non-collar groups for total number of impacts with various g-force cut-off (>20, >50, >100 g), total g-forces experienced (>20 g), and g-force per impact (>20 g).

In this analysis, the DTI measures (FA, RD, AD, and MD) at a given voxel were modeled as a function of sequence (order of treatment assignment), period (mid-season or post), and treatment (collar or no collar). Initially, group differences at a given time point (mid-season or baseline) were assessed using an independent two-sample *t*-test. This was followed by a within-group longitudinal change analysis between the two time points. For each subject within both the collar wearing group and the non-collar wearing group, a difference map between the two time points was calculated and the statistical significance and a paired *t*-test was used to assess the longitudinal change for each group. Finally, we used an independent two-sample *t*-test to assess whether the interaction between group and time was significant by comparing the longitudinal change between the two groups. The threshold-free cluster enhancement (TFCE) approach was adopted and the multiple comparisons were accounted for via family-wise error correction at *p* < 0.05 level. Correlation between the change in DTI measures and reaction time and memory composite scores as well as linear acceleration (*g*-force) and number of hits was examined using Pearson correlation at each voxel level. The TFCE approach and multiple comparison correction were again used in the correlation analysis as described relative to the group comparisons. The correlation between RD and the BNA Change score was examined using a Pearson correlation. The difference between BNA measures in the non-collar and collar groups was assessed using a Wilcoxon rank-sum test. Finally, a repeated measure ANOVA with Bonferroni correction was used to evaluate the ultrasound measures of Jugular vein dilation in response to collar wear.

## Results

### Jugular Vein Dilation in Response to Collar Wearing

The dilating action of the collar on IJV surface area was measured twice by ultrasound. There was a significant response in the IJV measured superior to the collar [*F*(2.26) = 55.37, *p* < 0.001]. Follow-up comparisons revealed that there was a significant increase in IJV dilation after both trials of collar wearing (M ± SD = 4.71 ± 0.33 mm) in the first trial and (4.61 ± 0.37 mm) in the second trial when compared to the test without collar (1.70 ± 0.23 mm; *p* < 0.001).

### Head Impact Surveillance

**Figure 4:**
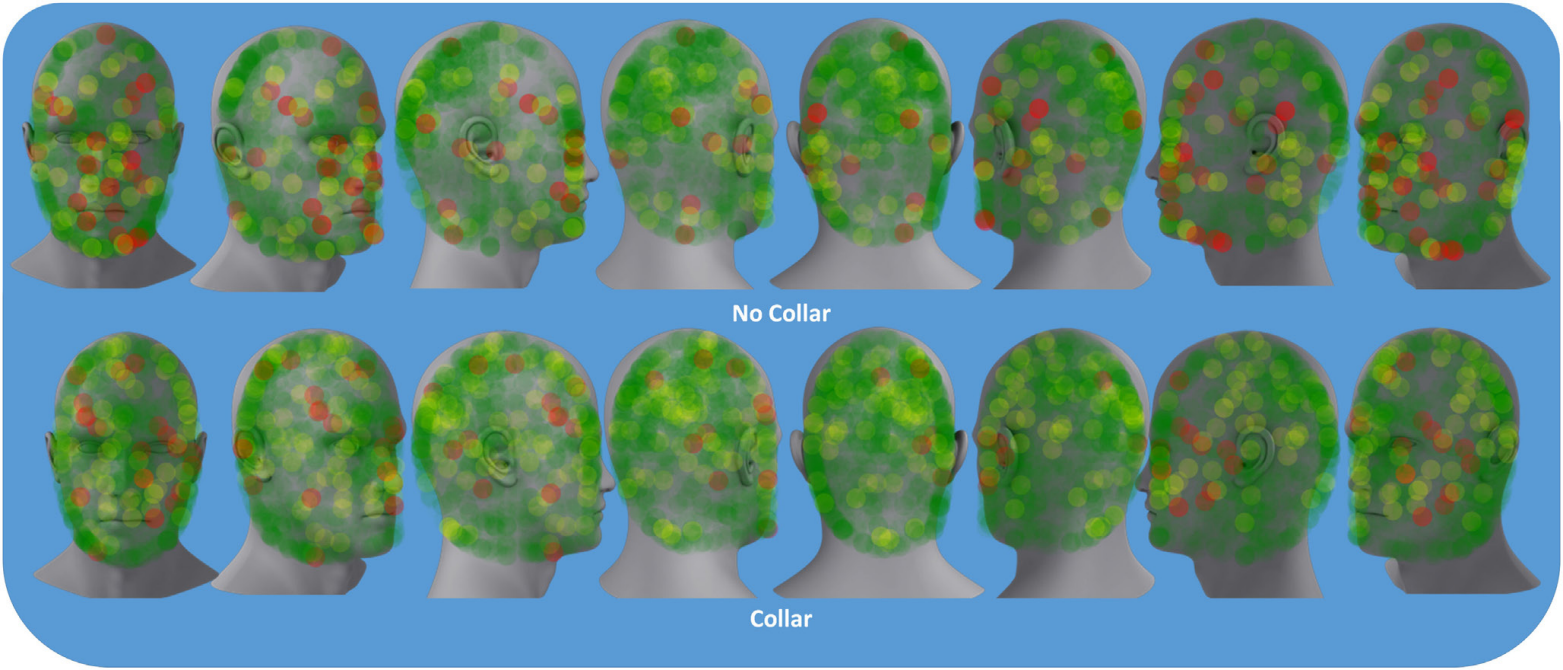
**Colored dots represent location of accelerometer measured the linear accelerations above 20 g (green), 50 g (yellow), and 100 g (red) sustained by either the collar or no-collar groups during the pre- to mid-season time period**.

Descriptive results of head impact exposures for each of the study groups are presented in Table 1A–C. The collar group experienced significantly more collisions, on average (linear acceleration threshold >50 g; *p* = 0.024) and trended toward increased average number of collisions (linear acceleration threshold >20 g; *p* = 0.052) and larger total accrued g-forces (*p* = 0.054) relative to the no-collar group during the first half season investigation period. There were no significant group differences with linear accelerations exceeding 100 g (*p* = 0.415) with no significant group differences in the average linear acceleration per collision above 20 g (*p* = 0.639) during the first half season investigation period. Figure 4 presents the location of accelerometer measured linear accelerations exceeding 20, 50, and 100 g thresholds, accumulated over the pre to mid-season phase of the investigation.

**Table 1:** **(A)** Average (±SD) # of hits at impact level; **(B)** average (± SD) of total g-forces experienced above impact level; **(C)** average g-force of hits above impact level.

	Group	Mean	SD
**A**
20 g	Collar	166.00	52.85
	No collar	117.50	35.73
50 g	Collar	23.57	11.18
	No collar	16.63	7.71
100 g	Collar	3.57	3.95
	No collar	4.13	3.00
**B**			
20 g	Collar	6311.52	1812.67
	No collar	4453.62	1621.84
50 g	Collar	1657.93	981.13
	No collar	1364.18	707.95
100 g	Collar	414.78	463.93
	No collar	565.94	413.86
**C**			
20 g/Collision	Collar	38.34	4.32
	No collar	37.40	3.30

### Longitudinal DTI Changes and the Effect of Collar Wearing

**Figure 5:**
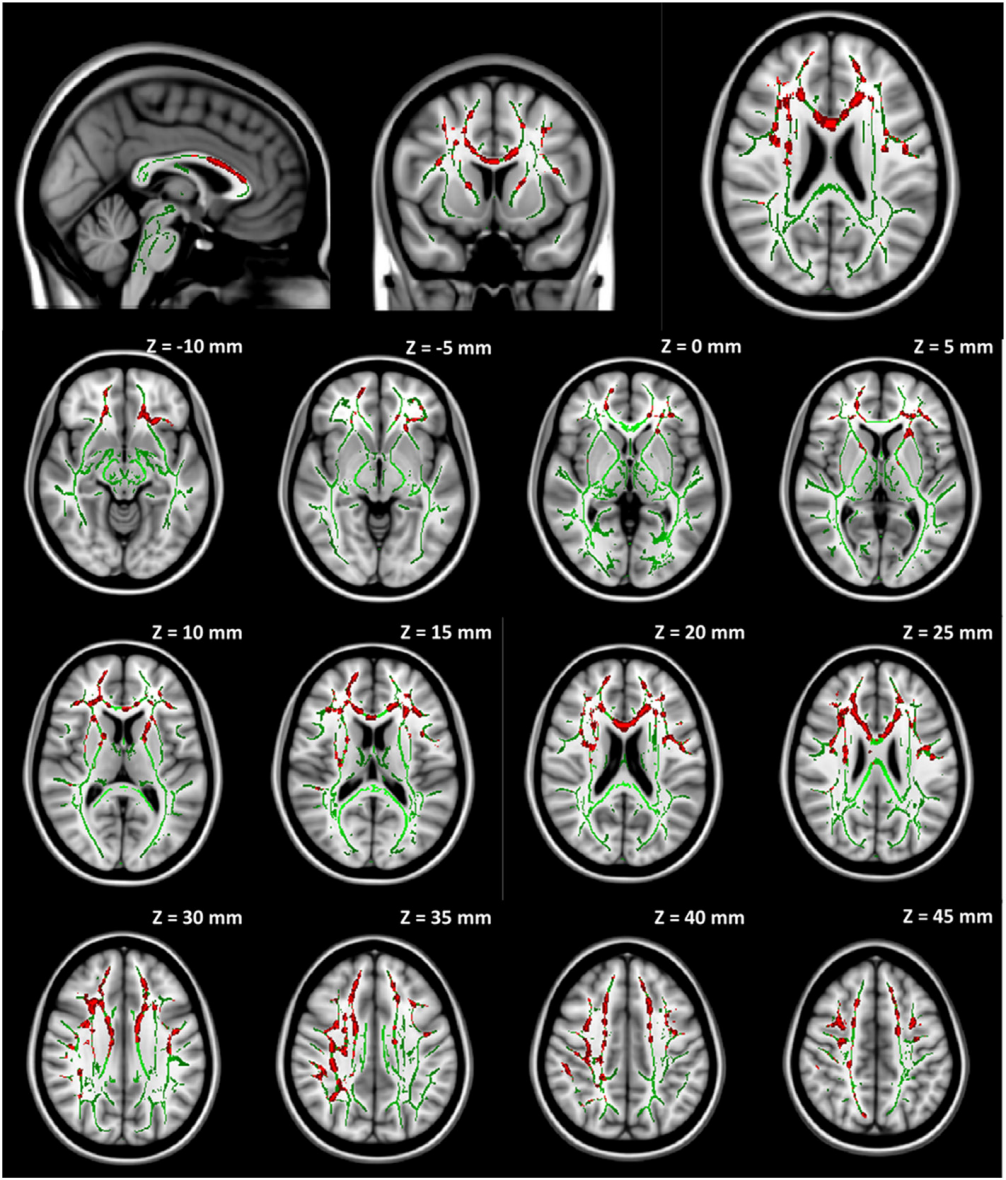
**Increase in MD from pre- to mid-season in non-collar group**. The red regions represent areas with statistically significant longitudinal changes at *p* < 0.05 level (FWE corrected). The red regions are thickened to improve visual display.

**Figure 6:**
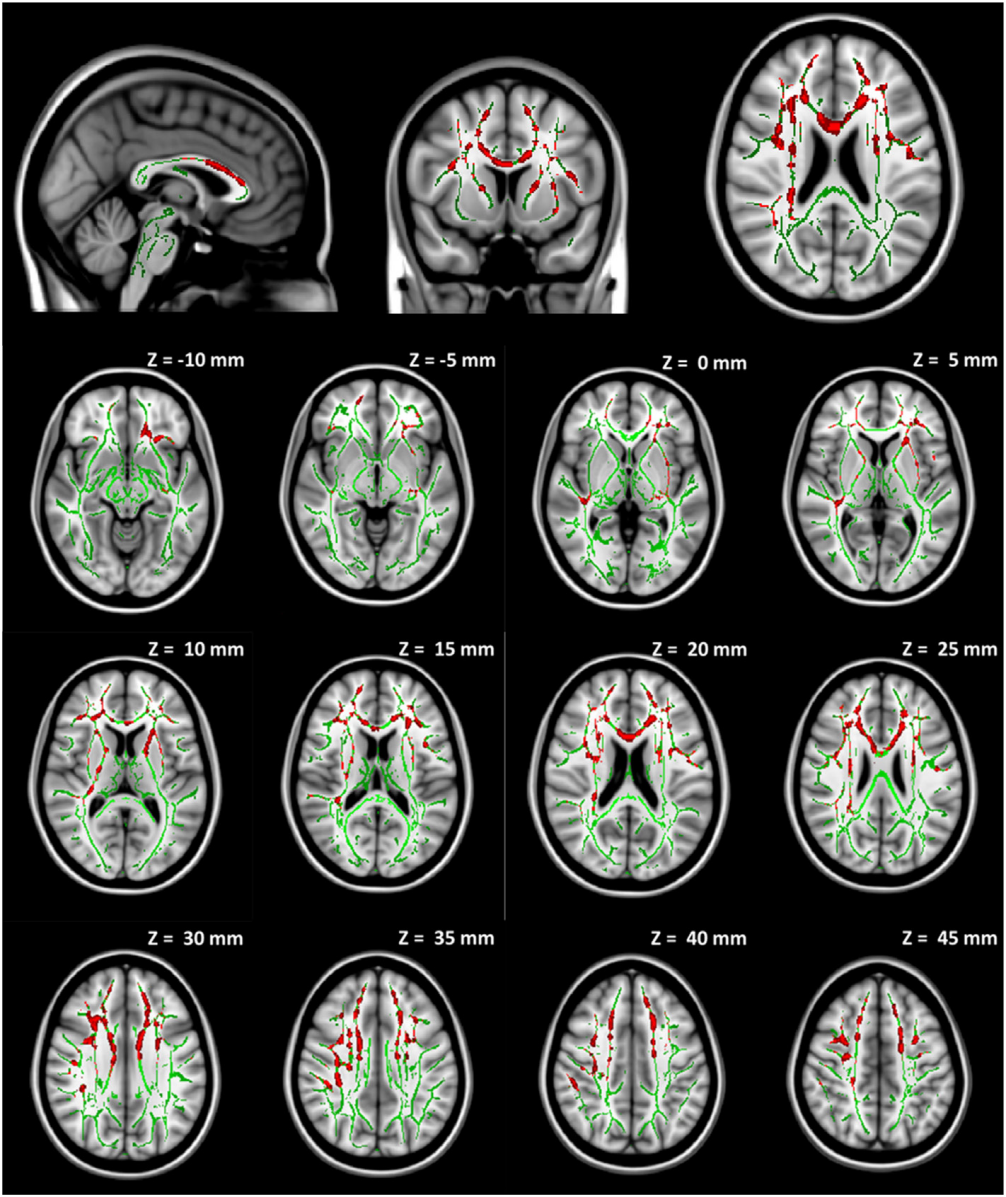
**Increase in RD from pre- to mid-season in non-collar group**. The red regions represent areas with statistically significant longitudinal changes at *p* < 0.05 level (FWE corrected). The red regions are thickened to improve visual display.

No significant group difference was found in any DTI measures at baseline between the seven participants initially assigned to the collar group and the seven participants initially assigned to the non-collar group. Longitudinally, no significant change was found in any DTI measures between pre- and mid-season in the collar group. In the non-collar group, significant increases in MD and RD from pre- to mid-season were found in extended WM areas (*p* < 0.05, FWE corrected), including (mostly bilateral) the corpus callosum (involving both the body and the genu), anterior, superior, and posterior corona radiata, anterior and posterior internal capsule, external capsule, superior longitudinal fasciculus, superior, middle, and inferior frontal gyrus WM, superior marginal gyrus WM, and superior parietal lobe gyrus WM (Figure 5: MD; Figure 6: RD). No significant longitudinal change was found in FA or AD in the non-collar group (*p* > 0.05).

**Figure 7:**
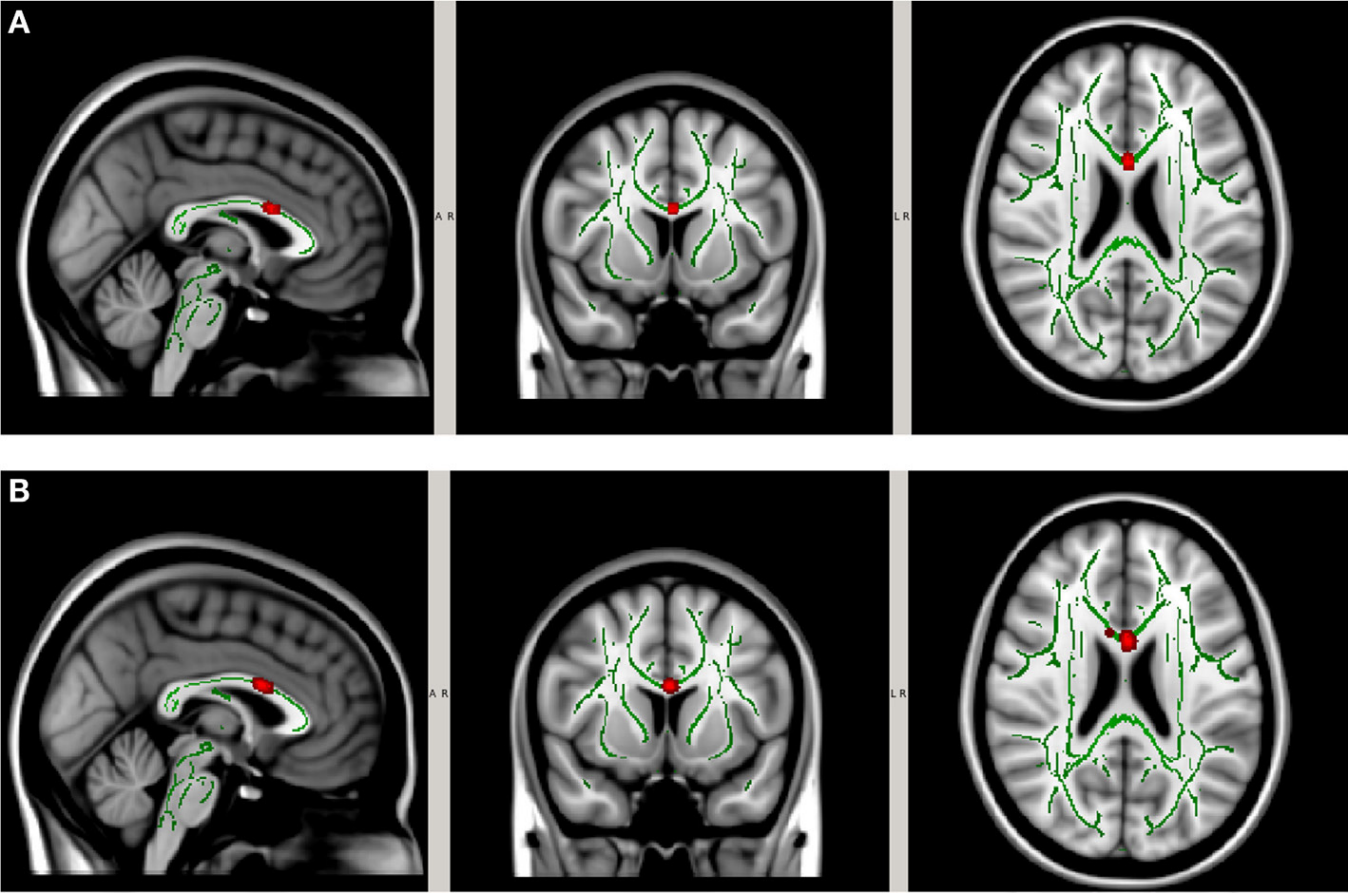
**Increase in MD (A) and RD (B) from pre- to mid-season in non-collar group after accounting for longitudinal changes in collar group**. The red regions represent areas where the increase of DTI measure in the non-collar group was significantly larger than that in the collar group, *p* < 0.05 level (FWE corrected). The red regions are thickened to improve visual display.

In order to account for potential confounding effects from factors other than the collar, we used the collar group as a control specifically we tested whether the longitudinal DTI change between pre- and mid-season observed in the non-collar group persisted after the pre- and mid-season change in the collar group was taken into consideration. As shown in Figure 6, the increase in MD (Figure 7A) and RD (Figure 7B) from pre- to mid-season in the non-collar group was significantly higher than that of the collar group in the body of corpus callosum. Descriptive statistics of MD and RD values in the two study groups within the region with significant between-group difference of pre- vs. mid-season change (i.e., “hot areas”) are presented in Table 2. The group differences of FA and AD change were also tested but no statistically significant result was found in these comparisons.

**Table 2:** **Descriptive statistics of MD and RD values in the two study groups within the region with significant between-group difference of pre- vs. mid-season change as shown in Figure 7**.

		Minimum	Median	Maximum	Average	STD
Collar baseline	MD	0.000732	0.000817	0.000894	0.000809	0.000056
Collar mid-season	MD	0.000675	0.00076	0.000897	0.000763	0.000072
No collar baseline	MD	0.000587	0.000785	0.000845	0.000760	0.000089
No collar mid-season	MD	0.00077	0.000839	0.000927	0.000843	0.000062
Collar baseline	RD	0.000466	0.000536	0.000586	0.000525	0.000044
Collar mid-season	RD	0.000392	0.000433	0.00058	0.000447	0.000062
No collar baseline	RD	0.000329	0.000417	0.000507	0.000426	0.000062
No collar mid-season	RD	0.000451	0.000525	0.000554	0.000511	0.000037

*Note that the DTI values represent *post hoc* data extracted from the brain regions with significant statistical significance in the test of within-group pre- vs. mid-season change (or in the test of between-group difference of pre- vs. mid-season change). These were not the data used in the statistical analysis that led to the findings as shown in Figures 5–7*.

### EEG Outcomes

**Figure 8:**
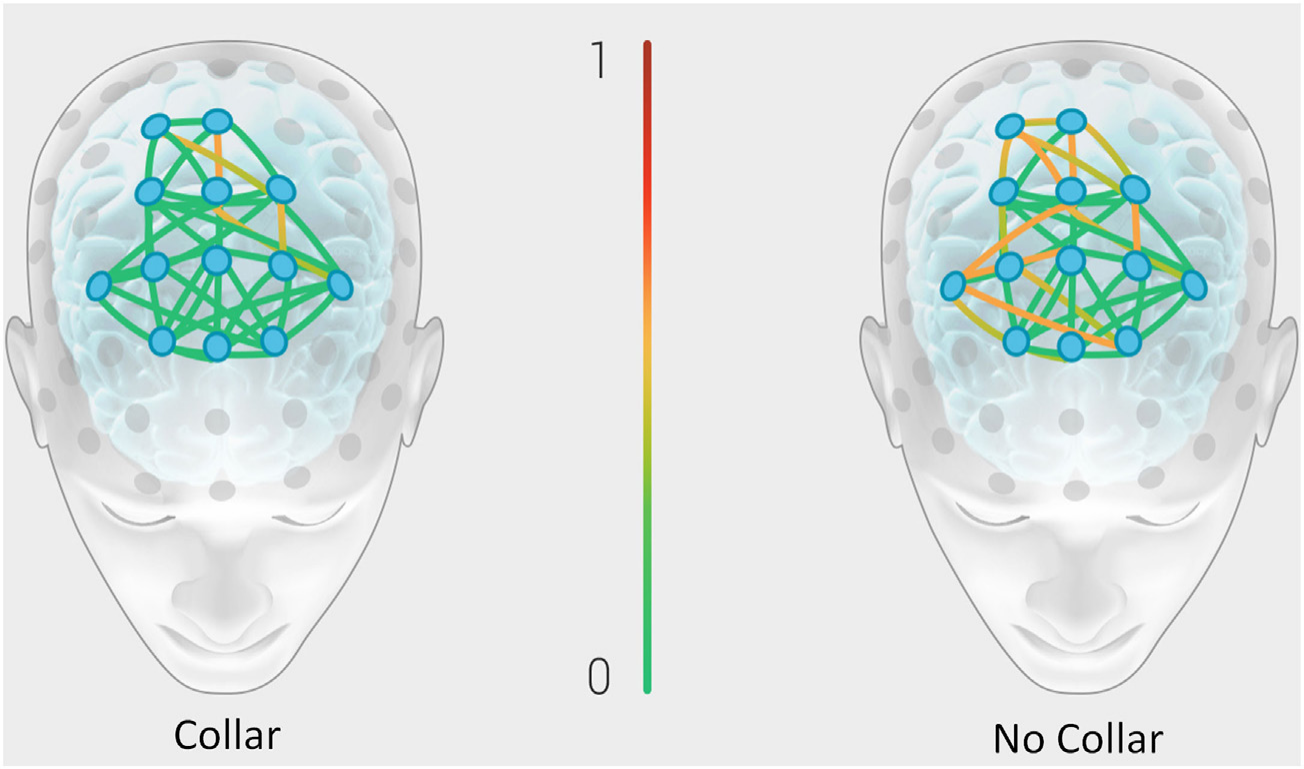
**Representation of the group mean for absolute value of the change in the BNA synchronization score between baseline and mid-season visits in the non-collar group vs. the collar group (BNA change score)**. Heat map imaging highlight increased change as the color moves away from green (0 = no change) to red (1 = high change).

Figure 8 represents the group mean for absolute value of change in the BNA synchronization score between baseline and mid-season visits in the non-collar group vs. the collar group (BNA change score) within each event pair of the RBNM. Heat map imaging highlights increased change as the color moves away from green. The BNA change score in the collar group remained relatively stable in comparison to the non-collar group (Figure 7). Similar to the DTI findings, the increased change in BNA score in the non-collar relative to the collar group was statistically significant (*p* = 0.006). Interestingly, the BNA change score in the non-collar group significantly correlated with the pre- to mid-season longitudinal RD changes (*r* = 0.76; *p* = 0.04).

## Discussion

This study examined the potential of the jugular vein impedance to modify the intracranial fluid dynamics and mitigate alterations to brain structure and function following head impact exposure. The collar device developed for evaluation is a novel, yet simple, approach for the internal prevention of brain movement when the head is exposed to concussive blows. The intervention under investigation was found to prevent alterations to WM integrity and brain network neurophysiology following head impact exposure. Importantly, the athletes tolerated that physiological change during competition and did so without subsequent anatomical or neurophysiological impairments.

Previous neuroimaging and outcome studies have reported significant changes in imaging biomarkers and the correlation of these biomarkers with cognitive outcomes after only one season of participation in contact sports with repetitive sub-concussion head impact (33). Consistent with the previous findings, but within only a half season, we identified significantly larger increase in MD and RD in the non-collar group than the collar group in the corpus callosum. The FA, MD, AD, and RD measures are standard DTI measures that have been used extensively in studying WM integrity in various neurological disorders/diseases (34). The direction of change in these measures are often interpreted and related to different injury mechanisms. Most commonly, decreased FA driven by the increase of MD and RD is often related to compromised myelin sheath or axonal membrane damage, while an increased FA accompanied by increased AD and decreased RD is often interpreted as the result of increased compression on WM tracts. In the current study, we found increased MD and RD from pre- to mid-season in the corpus callosum, which is suggestive of the initial sign of axonal and/or myelin degradation in the non-collared group. It should be noted that corpus callosum is the largest WM structure with extensive myelinated axons supporting interhemispheric information transferring. Altered WM integrity in the corpus callosum has been frequently reported in the TBI literature (35–37) and appears related to deficits in memory, attention, motor, and executive function, further resulting in worse clinical outcomes, greater severity in post-concussive symptoms, and other neuropsychological outcomes (35, 38–46).

Previous studies of jugular vein compression indicated no alterations in O_2_ uptake or glucose metabolism to any portion of the brain before or after jugular vein impedance (47). In a MR venography study, there was a significant increase in brain volume in the dural sinuses with the compression collar in place (22) and this volume increase normalizes as expected acutely following collar removal (22). It is theorized that these changes, although minimal, would translate to diminished brain motion within the cranial vault and, hence, less traumatic effects of sheer and rotational forces during competition (9, 48). It should be noted that these previous studies were conducted in the supine position and may not directly translate to sport. In the current study, athletes exhibited a physiologic response, in the upright position, to collar compression as hypothesized, and evidenced by IJV dilation assessed via ultrasound examination. While the mechanism(s) through which IJV distention translates directly to competition, or the extent of intracranial pressure and volume changes during competition are uncertain, the benefits of both anatomical and neurophysiological protection afforded by the collar in the current study are salient.

The collar device used in the present study did not negatively affect brain structures grossly or global cerebral function, as evidenced by MR imaging and EEG measures. The current results indicate, despite being subjected to similar head impacts, a change in WM integrity occurred only in the non-collar group. While it may be premature to reach a conclusion for the ultimate nature and significance of the finding, the alterations seen in the present study are congruent with prior studies to be a WM biomarker and possible injury (34, 49). Accordingly, the existence of these alterations in the non-collar group appears to reflect potential brain injury or long-term outcomes compared to the collar group.

In recent years, ERPs have been utilized as neurophysiological correlates of mild TBI (50) and as an objective index for chronic cognitive dysfunction associated with concussion (51, 52). In addition to anatomic changes demonstrated by DTI, we found larger electrophysiological changes between pre-season and mid-season in the non-collar group compared to the collar group (as evidenced by the BNA Change score – the absolute value of the change in the BNA synchronization score between pre-season and mid-season; Figure 7). It is possible, and even likely, that BNA is sensitive to the magnitude of brain injury and collar-based protection observed in the present study. Importantly, the BNA system has previously revealed differences between concussed athletes and healthy controls performing a cognitive task at 1 week post injury (31). Existing findings indicate that a network deficiency is involved in concussion (53, 54) and, thus, the consequences of mild TBI on the structure and function of large-scale brain networks is now becoming an extensive area of investigation.

The combination of EEG and DTI (40, 55–58) makes it possible to examine the relationship between functional and structural connectivity. The majority of previous studies that focused on structure–function associations utilizing EEG in task context have quantified the quality of signal transfer between the hemispheres using the interhemispheric transfer time (IHTT) or interhemispheric signal propagation (58). At rest, EEG–DTI studies indicated a relationship between interhemispheric coherence in the alpha band and DTI measures, thus allowing assessment of the efficiency of trans-regional communication (58). Structural alterations to WM tracts in concussion may, therefore, be related to reduced EEG phase synchrony between brain regions (59). The correlation between the BNA Change score and the difference in RD seen in our study may be associated with a temporal synchronization between brain regions that might have become impaired as a result of structural damage in their associated networks. Furthermore, this relationship between change in corpus callosum integrity and changes in network dynamics is congruent with other’s finding in young patients with moderate-to-severe TBI (60). Future research is warranted to determine if the BNA changes noted in no-collar group are associated with functional or quality of life measurements to help show if protection from change identified in the collar group is beneficial or not.

In the current investigation, functional and anatomical changes were identified in the participants without clinical concussive-type symptoms. There is emerging evidence that subconcussive blows may also result in brain injury. A study utilizing DTI pre-and post-season on high school athletes (football and hockey) found changes in WM in the asymptomatic athletes with subconcussive blows (61). These WM changes were three times greater than those seen in non-athletic controls (61), and similar changes have been shown in soccer and ice hockey athletes (62). These WM changes could remain after 6 months of rest from all activity (63). The full understanding of the effect of the subconcussive impacts remains an open question for medical professionals. Many contend that sub-concussions will lead to long-term detrimental effects functionally (15). Therefore, it is even more imperative that prevention strategies move toward promising technologies designed to protect the individual via an internal mechanism, as vetted in this study.

It should be stressed that accelerometers mounted in the helmets allow us to quantify head impact kinematics – including both linear acceleration and angular velocity – and commonly serve as a proxy for assessing the inertial behavior of the brain within the skull during an impact or collision (64). While head impacts have not been associated with short-term clinical measures of neurologic function (65), the current study results indicate that DTI and EEG provide sensitive measures to neuroanatomical and electrophysiological events following collision sport. Interestingly, despite the similarities in accumulated head impacts, the collar group experienced no statistically significant changes in each of the four DTI measures. This further highlights that the collar-induced jugular impedance may have attenuated the effects of head impact and may actually mitigate the concomitant neurological changes that are known to occur after subconcussive and concussive events (61).

The authors do acknowledge that the changes reported in the current study could be potentially explained by other factors, such as physiologic responses to other stresses in addition to trauma, developmental changes of the immature brain, genetics and/or other susceptibilities. The utilization of controlled longitudinal study design mitigates, but does not eliminate, the risk of these potential confounding variables. It is also acknowledged that physical activity and concussive events outside of hockey participation were not monitored although the exposure to collision forces during games and practices were closely monitored. In association with the current study, we explored the potential physiological effects of exercise on DTI measures in a sub-sample of matched athletes from the same school competing in track and field. The track athletes were assessed using identical MR sequences to current sample of hockey periods over a similar time period (Data not shown). The exploratory results indicated no significant changes in DTI measures in the track athletes as well as no group differences (collar vs. track) between the DTI outcome measures.

While the existing evidence and current study results indicate the collar approach as both safe and likely effective at mitigating the effects of concussive impacts, future research is warranted to determine the long-term effects of playing sport with mildly increased cerebral blood volume. Future research is also warranted to evaluate the effects of potential counter regulatory responses (decreased IJ distention over time or evidence of accommodation) in response to collar wear. This study was limited by the small sample size. Although we have carefully corrected for the multiple comparisons statistically to minimize the potential type I error, a larger scale study is warranted to corroborate the current findings. In addition, despite the 98.3% compliance rate in the first half of the season, the limited compliance of collar wearing in the second half of the season (in those athletes who were in the non-collar group in the first half of the season) requires further investigation. In particular, future efforts to better understand compliance, including the use of ITT statistical analyses would enhance related knowledge associated with the actual efficacy of the collar to ameliorate changes in WM microstructure following head impacts associated with competitive hockey.

## Conclusion

The current prospective, longitudinal analysis demonstrates statistically significant differential effects in brain structure and function from head impact. These data indicate that a mild jugular vein compression device may ameliorate the detrimental effects of collisions and resultant alterations in WM microstructure that are associated with brain injury in sport. The novel approach of this investigation and the early evidence, indicating a potential protection from WM alterations in response to head impacts, may help drive future efforts to prevent sports-related TBI.

## Author Contributions

GM contributed to study design, manuscript development, data analysis, and final review. WY contributed to study design, manuscript development, data analysis, and final review. KF contributed to study design, manuscript development, and final review. DS contributed to study design, manuscript development, and final review. MA contributed to study design, manuscript development, data analysis, and final review. AR contributed to study design, manuscript development, and final review. JL contributed to study design, manuscript development, and final review. AK contributed to study design, manuscript development, and final review. JK contributed to study design, manuscript development, data analysis, and final review. MW contributed to study design, manuscript development, and final review. ST contributed to study design, manuscript development, and final review. CD contributed to study design, manuscript development, and final review. JA contributed to study design, manuscript development, and final review. PG contributed to study design, manuscript development, and final review. AG contributed to study design, manuscript development, and final review. JC contributed to study design, manuscript development, and final review. WM contributed to study design, manuscript development, and final review. JM contributed to study design, manuscript development, and final review. DK contributed to study design, manuscript development, and final review.

## Conflict of Interest Statement

DS is the pioneer of the Q-Collar approach and has financial interest in the results of the current research. AR, MW, and AG have financial conflicts of interest with ElMindA technology. The remaining coauthors declare that the research was conducted in the absence of any commercial or financial relationships that could beconstrued as a potential conflict of interest.
